# Effect of physical exercise on sleep quality of college students: Chain intermediary effect of mindfulness and ruminative thinking

**DOI:** 10.3389/fpsyg.2022.987537

**Published:** 2022-09-26

**Authors:** Jun Ye, Xuemei Jia, Junjie Zhang, Kelei Guo

**Affiliations:** ^1^College of Physical Education, Huaibei Normal University, Huaibei, China; ^2^Sports Department, Capital University of Physical Education and Sports, Beijing, China; ^3^School of Physical Education and Health, Zhaoqing University, Zhaoqing, China

**Keywords:** college students, physical exercise, sleep quality, mindfulness, ruminative thinking

## Abstract

The Physical Exercise Rating Scale, Mindfulness Attention Awareness Scale (MAAS), Ruminative Thinking Scale (RRS), and Pittsburgh Sleep Quality Index (PSQI) are used to conduct the questionnaire among a sample of 1,006 college students (average age = 19.95 years, SD = 1.86 years) to figure out whether there is any correlation between physical exercise and sleep quality in college students as well as how the mechanism of mindfulness and ruminative thinking plays a role in them. For data analysis, the Harman single-factor test was used; for the common method deviation test, Pearson’s correlation analysis, and the mediating effect tested by using the bootstrap method were carried out. Results: (1) Overall, 34% (PSQI ≥ 8) of college students’ sleep quality is poor. (2) The PSQI is positively correlated with ruminative thinking, while both are negatively correlated with the level of physical exercise and mindfulness; the level of physical exercise is positively correlated with the level of mindfulness. (3) Physical exercise can significantly negatively predict sleep quality (β = −0.08, *P* < 0.05), significantly positively predict mindfulness (β = 0.12, *P* < 0.001), and significantly negatively predict ruminative thinking (β = −0.07, *P* < 0.05). When participate in physical exercise, mindfulness, and ruminative thinking enter the regression equation at the same time, only mindfulness and ruminative thinking can predict sleep quality(β = −0.15, *P* < 0.001) significantly negatively predicted sleep quality and ruminative thinking (β = 0.22, *P* < 0.001) significantly positively predicted sleep quality, while physical exercise (β = −0.04, *P* > 0.05) had no significant predictive effect on sleep quality. (4) After controlling for age and gender, physical exercise appears to have an impact on sleep quality of college students through the independent intermediary role of mindfulness and ruminative thinking and the chain intermediary role of mindfulness and ruminative thinking, with a total mediating effect value of −0.007. This study reveals the relationship between physical exercise and sleep quality, as well as its mechanism, thus guiding college students to actively participate in physical exercise. It also provides corresponding suggestions to improve sleep quality as well as physical and mental health in college students.

## Introduction

As a professionally cultivated new force of future society development, it is important to include the proper development of physical and mental health as an important prerequisite for learning and progress in college students. However, sleep quality of college students is getting worse, which hinders the development of their physical and mental health (Ghrouz et al., 2019; Shen, 2020). The sleep quality of college students in China has been gradually deteriorating in recent 20 years, suggesting that scientific sleep guidance should be given to college students (Fang et al., 2020). In recent years, it has been found that some college students are troubled with poor sleep quality in different regions of China. For example, the detection rate of sleep problems among 5,267 freshmen and sophomores in three universities in Shanghai is as high as 55.0% (Ye et al., 2019); the detection rate of poor sleep quality among 1,288 sophomores at Tibet University is 20.5% (Gao and Li, 2021); the detection rate of poor sleep among 6,224 freshmen and sophomores in four higher vocational colleges in Zhengzhou is as high as 52.81% (Wang and Li, 2020); and the detection rate of low sleep quality among 2,767 college students in Chenzhou city, Hunan Province, is 40.2% (Xi et al., 2018). Previous studies have shown that poor sleep quality may lead to fatigue, daytime sleepiness, depression, inattention, memory deterioration, and other phenomena (Wang and Wang, 2018). Since sleep quality is closely related to physical and mental health, as well as the learning growth of college students, sleep quality and its influencing factors have attracted extensive attention and research (You et al., 2020; Pang et al., 2021; Zhang et al., 2021).

The research on the impact of physical exercise on sleep quality has been relatively mature. One of the self-help therapies for people with sleep disorders is physical exercise (Zhang et al., 2016). Previous studies have shown that physical exercise relieves stress and negative emotions by secreting serotonin and dopamine, and promotes mental health (Archer et al., 2014). The reduction of psychological stress and negative emotions helps improve sleep quality (Zhao et al., 2020). Although there have been a lot of studies on the influencing factors of sleep quality, recent studies have focused more on depression, stress influence on anxiety, and other factors (Chen and Wu, 2021; Gao and Li, 2021; Ge Dandan et al., 2021; Pang et al., 2021; Zhang et al., 2021; Zhao et al., 2021), the research on the internal mechanism and intermediary role of physical exercise and sleep quality has not been fully revealed. Studying the effect of physical exercise on the sleep quality of college students and the mediating relationship between mindfulness and ruminative thinking not only enriches the research scope at the theoretical level of the influencing factors of college students’ sleep quality but also provides educators and college students with empirical evidence at the practical level from the perspective of exercise psychology that to strengthen physical exercise and to improve sleep quality.

### Physical exercise and sleep quality in college students

From the perspective of the exercise psychology theory, physical exercise can exert a positive impact on sleep quality of college students. A previous study found that compared with students with less physical exercise, students with a large amount of physical exercise have better sleep quality (Deng et al., 2018). A pilot study investigated whether routine physical activities of sedentary female students can improve their sleep (Hurdiel et al., 2017). A heart rate monitor was used to control the intensity of exercise, and the results showed that the sleep quality of the physical activity group was improved along with more intense exercise. Gong et al. (2019) conducted empirical research through the intervention of yoga and aerobic exercise among anxious female college students, and the results showed that exercise intervention can improve not only the sleep quality of college students but also the negative emotion level of anxious female college students. Other studies also pointed out that aerobic training and aerobic training combined with resistance training can inhibit excessive arousal, regulating negative emotions such as anxiety and tension, and improve sleep quality (Yang, 2020). The study also found that resistance training can promote skeletal muscles to release IL-6 for body immune regulation, which improves sleep quality to a certain extent. Based on studies mentioned previously, this study puts forward hypothesis 1: College students’ physical exercise has a significant positive impact on sleep quality.

### The mediating role of mindfulness

Kabatzinn (1996) pointed out that mindfulness is a kind of mood that accepts and focuses on the present moment without blaming or criticizing any issues. Studies have shown that physical exercise can positively predict individual mindfulness (Ulmer et al., 2010). Xu (2020) believes that physical exercise is very similar to mindfulness in attention style and attitude, and improving the enthusiasm for physical exercise is conducive to the improvement of the mindfulness level. Some studies also found that with the increase in exercise intensity, mindfulness feeling becomes more obvious (Cox et al., 2016). Nowadays, the protective role of mindfulness in sleep quality has attracted extensive attention (Gong et al., 2016; Ding et al., 2020). The relevant studies found that mindfulness tendency is related to better sleep quality (Cramer et al., 2016), and intervention based on mindfulness can effectively improve sleep quality (Blake et al., 2016). Other studies have concluded that college students with a higher mindfulness level have better sleep quality and lower depression score by studying the effect of mindfulness on college students when they are in a negative state (Tengshan et al., 2017). Other studies have concluded that the life satisfaction of college students participating in the pilot intervention was significantly improved, and sleep problems were significantly reduced (Dvořáková et al., 2017). This explanation can be attributed to the beneficial role of mindfulness in reducing stress reaction and enhancing the emotional balance, which is the core element of regulating sleep (Keng and Tong, 2016). In the study of mindfulness training, the participants report that it was easier to sleep because they could remove distractions and clear their thoughts by focusing on breathing and relaxing the body. For example, when students learned to focus on the body through mindfulness training so that different parts of the body can feel relaxed, they were well prepared for effective and quiet sleep (Howell et al., 2010). Therefore, it can be inferred that an increase in mindfulness level can improve sleep quality. Research shows that college students have improved their mindfulness level by participating in Pilates and Taijiquan courses (Caldwell et al., 2010). The increase in mindfulness means the change of emotion and perceived pressure, which promotes sleep quality to a certain extent. Therefore, this study puts forward hypothesis 2: Physical exercise can affect sleep quality through the intermediary role of mindfulness.

### The intermediary role of ruminative thinking

Ruminative thinking is defined as a continuous, past-oriented mood that focuses on negative thinking and its causes as well as consequences (Nolen-Hoeksema et al., 2008). Through the investigation of college students, research shows that people are more likely to have ruminative thinking, which has negative effects (Yu et al., 2020). Physical exercise has a positive impact on ruminative thinking. Empirical studies have found that participants with ruminative thinking mostly after the stress source show stronger and sustained negative emotions than those who ruminate less. However, when participants complete an aerobic exercise before the stress source, this lingering negative emotion weakened. These results show that exercise can weaken the subjective emotional effects from subsequent stress source (Bernstein and McNally, 2017). Research shows that ruminative thinking tendency will increase and prolong physiological stress responses, including HAP adrenal (HPA) axis response (Puterman et al., 2011). The research results of exercise on ruminative thinking response show that individuals who maintain physical exercise in life can be free from the effects of ruminative thinking on HPA axis reactivity and acute stress recovery. Existing studies generally believe that ruminative thinking and PSQI (the higher the sleep quality index is, the worse the sleep quality is) are significantly positively correlated (Liu et al., 2017; Yang et al., 2018; Li et al., 2020; You et al., 2020). The appearance and duration of insomnia are always caused by continuous self-blaming and over-attention to negative issues and mood. The research shows that the more the ruminative thinking times per day, the shorter the total sleep time (Felder et al., 2018). Ruminative thinking will increase negative emotions. At the same time, the thinking mode of this negative coping strategy will weaken the control of negative information, and the continuous negative state will cause sleep problems and health hazards (Slavish and Graham-Engeland, 2015). At the same time, persistent sleep delay will lead to a vicious cycle of stress–sleep interaction, making insomnia change from short-term to chronic (Harvey, 2002). Sedentary participants had higher levels of self-reported ruminative thinking on stress sources. Individuals with a high ruminative thinking level are more likely to have sleep quality problems (Slavish and Graham-Engeland, 2015; Butz and Stahlberg, 2018). Therefore, lack of physical exercise is likely to lead to a high ruminative thinking level and affect sleep quality. This study puts forward hypothesis 3: Ruminative thinking plays an intermediary role in the impact of physical exercise on sleep quality.

### The chain intermediary role of mindfulness and ruminative thinking

The research shows that mindfulness is negatively correlated with ruminative thinking very obviously (Liu et al., 2017; Parmentier et al., 2019). Therefore, the frequency of ruminative thinking tends to decrease among people with high mindfulness traits (Jury and Jose, 2018). Interventions based on mindfulness have been found to be able to reduce negative ruminative thinking (Calvete et al., 2021). Some studies had monitored 45 participants for 42 days on the mindfulness intervention process, and it was found that the degree of ruminative thinking decreased significantly after 1 week and 1 month (Andreotti et al., 2018). Experimental research has been conducted and found that mindfulness cognitive therapy reduces depression and ruminative thinking in the experimental group (Foroughi et al., 2020). Mindfulness cognitive therapy encourages people under depression to observe their thoughts and feelings without judgment. They are also encouraged to regard that as normal psychological activities, rather than their own problems. This cognitive attitude toward depression can prevent negative thoughts from escalating to ruminative thinking. Research shows that mindfulness training is conducive to improving self-control ability of college students and reducing the ruminative thinking level (Cheng et al., 2020) so as to inhibit the negative impact of smartphone addiction on college students before going to bed, thus promoting their sleep and mental health. In fact, from the essence of mindfulness, college students with a high mindfulness level can easily focus on learning, rather than thinking about the past and worrying about the future, which can reduce ruminative thinking and improve the sleep quality of college students. Based on this, this study puts forward hypothesis 4: Mindfulness and ruminative thinking play a chain intermediary role between physical exercise and sleep quality.

## Materials and methods

### Participants

The stratified cluster sampling method was used to select five universities (including comprehensive universities, normal universities, and sports universities) in South China, central China, and North China, and two classes were randomly selected from each grade of each university to distribute questionnaires. A total of 1,006 valid questionnaires were collected (invalid questionnaires were excluded due to regular answers, lack of data, and other reasons), and the effective recovery rate was 94.9%, with 464 boys (46.1%) and 542 girls (53.9%), 26.62% in the first grade, 25.66% in the second grade, 27.7% in the third grade, and 20.02% in the fourth grade, with an average age of 19.95 (SD = 1.86) years.

The test was carried out with the consent of the college leaders, teachers, and the subjects themselves. The test was conducted collectively, emphasizing the principles of voluntary filling, data confidentiality and anonymous filling, and controlling the variables such as gender and grade of the subjects. In accordance with the Declaration of Helsinki, this study has been approved by the Institutional Review Board of College of Physical Education at Huaibei Normal University. This study takes about 5–10 min to complete all the questionnaires. In this process, all invited participants are voluntary and confidential.

### Measures

#### Physical Exercise Rating Scale

The Physical Exercise Rating Scale was adopted, introduced, and revised in Chinese (Liang, 1994). The scale measures the level of participation of exercisers by calculating the amount of physical exercise. It mainly investigates the amount of physical exercise the subjects performed in 1 month and tests the physical exercise of the subjects from three aspects: intensity, duration, and frequency. The score of exercise = exercise intensity × (score for each exercise time − 1) × exercise frequency. Each aspect is divided into five grades (1–5 points). The maximum score of exercise is 100 points, and the minimum score is 0 point. The evaluation standard of exercise amount: the small exercise amount is ≤19 points, the average amount of exercise is 20–42 points, and the large amount of exercise is ≥43 points. The aforementioned test is of high reliability and validity (Tang et al., 2009). The test-retest reliability of the scale is 0.82.

### Mindfulness Awareness Scale

The Mindfulness Awareness Scale (MAAS) was developed by Brown and Ryan (2003) and revised by Chen et al. (2012) to measure the mindfulness level based on the concept of “current attention and awareness.”. The scale is a one-dimensional structure with 15 items (such as “I may not be aware of some emotions until they last for a period of time”) with each item has a six-point score from 1 (often) to 6 (never). The higher the total score, the higher the mindfulness trait of the individual. The results of confirmatory factor analysis were as follows: TLI = 0.904, IFI = 0.918, CFI = 0.917, RMR = 0.067, and RMSEA = 0.077. Cronbach’s α index is 0.856.

#### Ruminative Responses Scale

The Ruminative Responses Scale (RRS) was revised, which includes three subscales include symptom rumination, forced thinking, and reflection, with a total of 22 items (such as “I often think why I am so unhappy”) (Han and Yang, 2009). The scale adopts a four-point scoring method: 1 means never or occasionally, and 4 means always or most of the time. All items are scored positively. The higher the score, the more serious the rumination thinking. The overall Cronbach’s α coefficient of the questionnaire is 0.95; the three-dimensional Cronbach’s α coefficient is between 0.88 and 0.94; the results of confirmatory factor analysis were as follows: χ^2^/df = 4.81, CFI = 0.92, TLI = 0.91, and RMSEA = 0.08.

#### Pittsburgh Sleep Quality Index

The Pittsburgh Sleep Quality Index (PSQI) was compiled and revised by some authors (Buysse et al., 1989; Liu et al., 1996). It aims to measure and evaluates the sleep quality of college students in the previous month. The scale consists of 18 self-assessment items, which can be divided into seven components: subjective sleep quality, sleep time, sleep efficiency, sleep disorder, hypnotic drugs, and daytime dysfunction. Each item is scored according to 0–3 points, and each component is accumulated as the total score of the PSQI. The higher the score, the worse the quality. Some research took a PSQI ≥ 8 as the standard to evaluate the quality of sleep (Liu et al., 1994). The scale has good reliability and validity as the scale α coefficient is 0.80. The results of confirmatory factor analysis were as follows: χ^2^/df = 2.04, RMSEA = 0.05, CFI = 0.98, NFI = 0.96, and GFI = 0.98.

### Statistical analyses

SPSS 26.0 and its plug-in process were adopted to input, process, and statistically analyze the relevant data of this study, which include descriptive analysis and Pearson’s correlation analysis to figure out the total score of the scale and its dimensions. The data were normalized by *Z*-score. In this study, only self-reported data were collected, which may cause common method bias. The bootstrap method was used for the mediation effect of inspection, not judged by *P*-value, but according to the (BootLLCI, BootULCI) judging whether the interval containing 0. If 0 is not included, the mediating effect is significant, while if 0 is included, it is not significant. In order to further improve the rigor of the study, the Harman single-factor test was used to test the deviation of the common method before data analysis. The results show that there are 14 factors with characteristic roots greater than 1, accounting for 57.89% of the total variance, and the variation explained by the first factor is 18.46%, indicating that there is no significant common method deviation (Zhou and Long, 2004).

## Results

### The descriptive statistics and correlation analysis of research variables

Based on the standard of a PSQI ≥ 8, the detection rate of poor sleep quality of college students was 34% (342 subjects). The descriptive statistics and correlation analysis of research variables, means, standard deviations, and correlation coefficients of each variable are shown in Table 1. The PSQI (the higher the sleep quality index, the worse the sleep quality) is positively correlated with ruminative thinking but negatively correlated with physical exercise and mindfulness; physical exercise is positively correlated with mindfulness but negatively correlated with ruminative thinking; and ruminative thinking is negatively correlated with mindfulness.

**TABLE 1:** Descriptive statistics of the main variables and their correlation analysis.

	*M*	SD	1	2	3	4
1. PE	19.70	16.07	1			
2. SQ	6.30	3.71	−0.06*	1		
3. MIN	54.29	12.26	0.12**	−0.24**	1	
4. RT	44.50	10.87	−0.10**	0.28**	−0.37**	1

*N* = 1,006. PE, physical exercise; SQ, sleep quality; MIN, mindfulness; RT, ruminative thinking.

*P < 0.05, **P < 0.01.

### Analysis of the mediating effect

The intermediate effect test was verified according to the statistical method of Wen and Ye (2014), and the data were sorted and analyzed by SPSS macro program process of SPSS 26.0 and Hayes (2013). Under the condition of controlling gender and age, the mediating effect was tested by estimating the 95% confidence interval (CI) of mediating effect through 5,000 sample sampling. The results show that (Table 2) physical exercise can significantly negatively predict sleep quality (β = −0.08, *P* < 0.05); significantly positively predict mindfulness (β = 0.12, *P* < 0.001), and significantly negatively predict rumination (β = −0.07, *P* < 0.05). When participate in physical exercise, mindfulness and rumination enter the regression equation at the same time, only mindfulness and rumination can predict sleep quality (β = −0.15, *P* < 0.001) significantly negatively predicted sleep quality and rumination (β = 0.22, *P* < 0.001) significantly positively predicted sleep quality, while physical exercise (β = −0.04, *P* > 0.05) had no significant predictive effect on sleep quality.

**TABLE 2:** Regression analysis of variable relationships.

Regression equation		Overall fit index			Significance of coefficient of regression		95% CI	
			
Outcome variable	Predictive variable	*R*	*R* ^2^	*F*	β	*t*	Bootstrap lower limit	Bootstrap higher limit
SQ	Gender	0.11	0.01	4.39	−0.01	−0.25	−0.57	0.44
	Age				0.09	2.95**	0.06	0.31
	PE				−0.08	−2.35*	−0.02	−0.002
MIN	Gender	0.12	0.02	5.09	−0.01	−0.32	−1.94	1.39
	Age				−0.02	−0.55	−0.53	0.30
	PE				0.12	3.45***	0.03	0.10
RT	Gender	0.38	0.14	42.07	−0.04	−1.28	−2.28	0.48
	Age				−0.005	−0.16	−0.37	0.31
	PE				−0.07	−2.06*	−0.06	−0.001
	MIN				−0.37	−12.51***	−0.38	−0.28
SQ	Gender	0.33	0.11	23.99	−0.002	−0.07	−0.50	0.47
	Age				0.09	3.00**	0.06	0.30
	PE				−0.04	−1.17	−0.02	0.004
	MIN				−0.15	−4.69***	−0.07	−0.03
	RT				0.22	6.77***	0.05	0.10

Bootstrap sample size = 5,000. Bootstrap 95% CI does not contain 0 value, indicating a significant coefficient.

*P < 0.05, **P < 0.01, ***P < 0.001.

**FIGURE 1:**
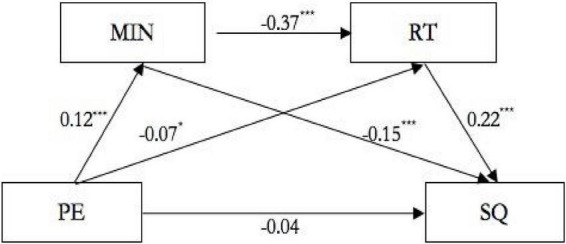
Pathway map of PE affecting SQ. *p < 0.05, ***p < 0.001.

By further testing the mediating effect (Table 3), the results show that the bootstrap 95% CI of the total indirect effect of mindfulness and rumination on physical exercise and sleep quality does not contain the index 0, indicating that mindfulness and rumination have a significant mediating effect on the impact of physical exercise on sleep quality. In total, three paths constitute this mediating effect: first, physical exercise → mindfulness → sleep quality, and the latter interval does not contain a value of 0, indicating that the indirect effect produced by this path is significant (the effect value is −0.003, accounting for 22% of the total effect value). Second, physical exercise → rumination → sleep quality, and the CI of the indirect effect does not include 0 value, indicating that the indirect effect produced by this path is significant (the effect value is −0.002, accounting for 18% of the total effect value). Third, physical exercise → mindfulness → rumination → sleep quality, and the CI of the indirect effect does not include 0, indicating that the indirect effect produced by this path is significant (the effect value is −0.002, accounting for 12% of the total effect value). The path of physical exercise on sleep quality is shown in Figure 1.

**TABLE 3:** Test of intermediary effect.

Path	Indirect effect value	BootSE	BootLLCI	BootULCI	Relative mediation effect (%)
Total indirect effect	−0.007	0.002	−0.010	−0.003	52.00
PE → MIN → SQ	−0.003	0.001	−0.005	−0.001	22.40
PE → RT → SQ	−0.002	0.001	−0.005	−0.0001	17.60
PE → MIN → RT → SQ	−0.002	0.001	−0.003	−0.001	12.00

## Discussion

### The influence mechanism of physical exercise on sleep quality

In this study, gender and age were used as control variables to test the effect of physical exercise on sleep quality and to analyze the internal mediating mechanism of mindfulness and rumination. It was found that physical exercise has a significant positive impact on sleep quality of college students, which is consistent with the previous research results (Zhao et al., 2020; Wang, 2021) that the intensity, time, and frequency of physical exercise will affect sleep quality. The sleep quality of college students is closely related to the degree of physical exercise. Few studies have been conducted to discuss the internal mediating mechanism of the impact of physical exercise on sleep quality. It is noteworthy that after introducing the two intermediary variables of mindfulness and rumination, the direct predictive effect of physical exercise on sleep quality is not significant. However, the two separate intermediaries of mindfulness and rumination and the chain of physical exercise → mindfulness → rumination → sleep quality mediators further affect sleep quality. This result shows that physical exercise of college students will not directly improve their sleep quality, and mindfulness and rumination are important factors in the relationship between them.

### Mediating effect of mindfulness

The research results show that physical exercise can affect the sleep quality of college students through a separate mediating effect of mindfulness. College students who actively participate in physical exercise are conducive to the improvement of their mindfulness level (Xu, 2020). The higher mindfulness level of college students means that they are more likely to adhere to physical exercise for a long time (Lentz and Brown, 2019) and to increase the duration of exercise (Robin et al., 2019). With the increase in exercise intensity, the mindfulness feeling of the body becomes more obvious (Cox et al., 2016). The improvement of the mindfulness level can inhibit negative state and improve life satisfaction of college students so as to significantly improve sleep quality of college students (Dvořáková et al., 2017; Teng et al., 2017). The finding that the quality of sleep can be improved by increasing the level of mindfulness through physical exercise is consistent with previous research results (Caldwell et al., 2010). For college students who actively participate in physical exercise, mindfulness is a positive factor for their mental health. A higher mindfulness level can improve individuals’ psychological adaptation. Therefore, the improvement of the mindfulness level is an important factor to promote college students who actively participate in physical exercise to improve their sleep quality.

### Mediating effect of ruminative thinking

The research results show that physical exercise can affect the sleep quality of college students through the separate mediating effect of ruminative thinking. First, participating in physical exercise can weaken lingering negative emotions and reduce the ruminative thinking level, which can improve the sleep problems of college students due to their high ruminative thinking level to a certain extent (Bernstein and McNally, 2017). Second, it was found through empirical research that sedentary individuals have faster and longer response to stress (Puterman et al., 2011). The delayed recovery of stress takes the form of faster initial increase, later peak, delayed recovery to baseline, and a higher self-reported rumination level of stress. Individuals with high a ruminative thinking level are more likely to have sleep quality problems, which is consistent with many previous studies. The results of the researchers are consistent (Slavish and Graham-Engeland, 2015; Butz and Stahlberg, 2018). For college students who lack physical exercise, rumination thinking is a risky factor for their mental health, and higher ruminative thinking tends to exert a negative impact on their sleep quality. Therefore, the high level of ruminative thinking is an important factor for college students who lack physical exercise and have low sleep quality.

### The chain intermediary of college students’ mindfulness and ruminative thinking

It is found that physical exercise has an impact on sleep quality of college students through the chain intermediary effect of mindfulness and ruminative thinking. First, the level of mindfulness can be improved through physical exercise, which can inhibit ruminative thinking and improve the quality of sleep. It is found through empirical study that among the subjects in the yoga and exercise groups, the mindfulness awareness score of the yoga group increased significantly, and the mindfulness acceptance score of the exercise group also increased significantly, but perceived stress and ruminative thinking were significantly reduced to the normal level (La Rocque et al., 2021). By training medical college students in MAP (Meditation and Aerobic Exercise for Brain Health), describing the benefits of meditation and other mindfulness exercises before each MAP session, participants reported a significant improvement in their quality of life and a significant reduction in rumination after 8 weeks of training (Lavadera et al., 2020). There are similar arguments in other studies (Curlik and Shors, 2013; Shors et al., 2014). Therefore, it can be concluded that physical exercise can improve the level of mindfulness and reduce the level of ruminative thinking and improve the quality of sleep. Previous studies have pointed out that maintaining physical exercise is closely related to high sleep quality, but few has discussed the further impact of internal psychological factors such as mindfulness and ruminative thinking on sleep quality. This study has answered this question from the perspective of physical exercise and mental health promotion, which is of great value.

### Practical effect

The results of this study have an important positive role in encouraging college students to actively participate in physical exercise. First, college students who lack physical exercise may have a higher level of ruminative thinking, resulting in the spread of negative emotions and endangering sleep quality as well as both physical and mental health. Therefore, college teachers should pay more attention to physical exercise and publicize its importance. Various forms of campus sports activities are suggested to be organized to cultivate the sports culture and atmosphere at campus and to guide college students to actively participate in physical exercise. Second, the chain intermediary effect suggests that physical exercise and high mindfulness level can reduce rumination and improve sleep quality. In addition, several experimental studies have mentioned that yoga, meditation, and aerobic exercise can be treated as exercise methods to improve the level of mindfulness (de Bruin et al., 2016; Lavadera et al., 2020; La Rocque et al., 2021; West et al., 2021). Therefore, yoga should be one of the compulsory courses among college physical education courses. In addition, college physical education teachers should be trained to learn meditation and breathing methods to teach students at the warm-up section of physical education courses, so as to improve the mindfulness level of college students.

### Limitations and future directions

The findings of this study have certain theoretical value and practical guidance but also have some limitations. First, this study is a cross-sectional study, the data were collected at once, and no effective follow-up study was conducted. But there is no continuity to establish the causal relationship in the future to increase the longitudinal follow-up or experimental intervention designs study, complete and comprehensive analysis of the entire development process and key points. Second, the design and distribution of the questionnaire were not complete, making the subjects to fill out the questionnaire with some concerns of catering to social acceptance factors. Moreover, due to the limitation of time and place, all data were collected using a self-report method, which made the objectivity of the data somewhat biased. Third, because there are many factors influencing the explanatory variables, the physical exercise analyzed in this study is only one aspect affecting their sleep quality, and the chain intermediary model made is not the only mediation model. In this study, only mindfulness and ruminative thinking are considered as intermediary variables. In fact, there may still be other intermediary variables such as perceived stress and extent of relaxation; therefore, the conclusion of this article is only a small part affecting the sleep quality, and there are many other explanatory and mediating variables worth being tested in the future.

## Conclusion

The research results show that physical exercise can significantly positively predict sleep quality. Mindfulness and ruminative thinking play a significant mediating role between physical exercise and sleep quality. There are three mediating paths: first, the separate mediating effect of mindfulness; second, the separate mediating effect of ruminative thinking; and third, the chain mediating effect of mindfulness and ruminative thinking. Therefore, physical exercise can not only improve the sleep quality of college students through the separate mediating effect of ruminative thinking but also improve sleep quality by improving the level of mindfulness and reducing the level of ruminative thinking. This result shows that physical exercise of college students will not directly improve their sleep quality, but mindfulness and rumination are important factors in the relationship between them.

Previous studies have pointed out that maintaining physical exercise is closely related to high sleep quality, but few studies have discussed the further impact of internal psychological factors, such as mindfulness and ruminative thinking on sleep quality. This study has answered this question from the perspective of physical exercise and mental health promotion. By studying the effect of physical exercise on the sleep quality of college students and the mediating relationship between mindfulness and ruminative thinking, it not only enriches the research scope at the theoretical level of the influencing factors of college students’ sleep quality but also provides educators and college students with empirical evidence at the practical level from the perspective of exercise psychology that to strengthen physical exercise and to improve sleep quality. This study can provide insights for effectively improving the sleep quality of college students, and the results have an important positive role in encouraging college students to actively participate in physical exercise.

## Data availability statement

The datasets presented in this study can be found in online repositories. The names of the repository/repositories and accession number(s) can be found in the article/supplementary material.

## Ethics statement

Ethical review and approval was not required for the study on human participants in accordance with the local legislation and institutional requirements. Written informed consent from the patients/participants OR patients/participants legal guardian/next of kin was not required to participate in this study in accordance with the national legislation and the institutional requirements.

## Author contributions

JY designed the study, collected and analyzed the data, and wrote the manuscript. XJ, JZ, and KG revised the manuscript. All authors contributed to the article and approved the submitted version.
